# Life Insurance Examining

**Published:** 1906-08

**Authors:** 


### EDITORIAL NOTES AND COMMENTS.

The Business office of THE JOURNAL-RECORD is No. 1007-1008 Century Building.

The Editorial office is 318-319-320 The Prudential.

Address all Business communications to Dr. M. B. Hutchins, Mgr.

Make remittances payable to THE ATLANTA JOURNAL-RECORD OF MEDICINE.

On matters pertaining to the Editorial and Original Communications address Dr. Bernard Wolff, Atlanta.

Reprints of original articles will be furnished at cost price. Requests for the same should always be made *on the manuscript*.

We will present, post-paid, on request, to each contributor of an original article, twenty (20) marked copies of THE JOURNAL-RECORD containing such article.

### LIFE INSURANCE EXAMINING.

We print elsewhere in this issue a statement of the insurance companies' side on the question of the free-tariff for life-insurance examinations.

This question is becoming pertinent and personal to members of the regular medical profession, and they must face the issue squarely. Already an old-line insurance company is endeavoring to secure examiners on a two-year contract on a scale of fees ranging from $3 to $10, with the probable end in view of forestalling action on the part of medical organizations fixing compensation for insurance examining.

It would be interesting to conjecture the result of signing this contract to a member of a regular medical body in the event of the passage of resolutions quite at variance with the terms of the contract. It would, at least, throw him in the awkward position of being forced either to commit a breach of contract, or to lose standing and become, in a way, a professional free lance. Although comparatively few county societies have as yet adopted resolutions mandatory in character, it requires but little forethought to perceive that many more will take similar action in the near future, and that the movement will be crystallized by formal resolution on the part of the State Association. This condition has appeared lately in Macon, where a highly esteemed member of the local fraternity from refusing to subscribe to the resolutions on insurance examinations adopted by the Macon (Bibb county) Medical Society was subjected to much inconvenience and embarrassment on account of severance of professional rapport, and came in for a large share of public sympathy as a victim of alleged persecution.

It is an awkward predicament in which to be placed, but one in which many members of the profession will doubtless be thrown as the movement gains ground, and it is well for every insurance examiner in the State to take due and timely notice and govern himself accordingly.

Without going into the merits of the fee-bill case, it is entirely evident that the insurance company and the medical profession are connected by a close bond of union, but with the very essential difference that the insurance company is fundamentally dependent upon the medical profession, while the medical profession looks upon the insurance company as a very considerable and acceptable, but by no means indispensable, source of financial gain. In fact, there are not a few examiners who chafe under the infliction of persistent and exacting insurance agents, and prefer to relinquish their appointment, however profitable, rather than further submit to the ineffable boredom of the position. Of course, this is not the usual condition, but every one who has done insurance work knows the humiliation that follows a lessening of self-respect at being pushed about like a pawn in the hands of a selfish, pernicious and irrepressible agent. If, on the other hand, the solicitor should find the doctor a somewhat difficult person, he may be able to carry his application to some other and more congenial examiner, but he can not avoid the doctor altogether. The doctor is a necessary adjunct to the agent's business, and his limitations are definite. The fowl must be weighed and felt over before he is plucked and devoured. Another feature of the discussion concerns the doctor as an economic factor in aiding the insurance company in carrying out the policy of retrenchment which has been forced upon it by investigation and the passage of laws. The doctor may view with polite indifference, even with a show of sympathy, the shrinkage in the size of the agent's commission under the present régime, but there is no reason why he should share the agent's misfortune. Should the doctor's practice diminish and the volume of his income lessen, would the insurance company lower his premium rate on that account, to form an adjustment to his waning fortune and aid him in economizing? We are told that a rule should work both ways. Does it do so in this case?

---

IT is with much regret we note the death of Fritze Schaudinn, M.D., who only a little more than a year ago attained world-wide fame by his discovery of the *spirocheta pallida,* or *treponema pallidum.*

His death occurred June 22, soon after he had accepted an important position in the Parasitologic Department of the Institute for Tropical Diseases in Hamburg. Septic infection followed a periproctitic abscess soon after his return from the International Medical Congress at Lisbon, where he was hailed as the discoverer of the micro-organism which causes syphilis. Of all the germs that have been brought forward as the causal factor of syphilis, the treponema pallidum is the only one which has stood the test of confirmation by other investigators than the discoverer.

Schaudinn was only thirty-six years of age, but was widely known before his discovery of the treponema pallidum, for his excellent, painstaking work in the field of the protozoa.

His death, so untimely and unexpected, is a distinct loss to the medical profession of the world. It is particularly sad that he should not have lived to see the general acceptance of his discovery and its utilization in the diagnosis of syphilis, and perhaps other practical advantages in the management of this disease that we are now unable to see.

---

DR. EDWARD GEDDINGS, one of the best-known physicians of the South, died at his country home near Augusta, June 25th, aged seventy-four. Dr. Geddings was the first American to be graduated from the University of Berlin, Germany, and has been intimately connected for years with the medical department of the University of Georgia.

---

DR. J. N. LE CONTE, after a year's study at the leading clinics of Europe, has returned to Atlanta, where he will devote himself to his specialty, diseases of the stomach and intestines. We welcome Dr. LeConte back to Atlanta, and wish him the success we feel sure he will attain. His offices are 522-3-4 Century Building.

---

ATTENTION is called to notice of competitive examination for position of assistant physician State Sanitarium on page xvi.

